# Resilience and adaptations—insights from Norwegian adolescents with pediatric-onset spinal cord injury

**DOI:** 10.3389/fresc.2025.1526431

**Published:** 2025-04-03

**Authors:** Wiebke Höfers, Kirsti Riiser, Vivien Jørgensen, Solveig L. Hauger, Kirsti Skavberg Roaldsen

**Affiliations:** ^1^ Department of Research and Innovation, Sunnaas Rehabilitation Hospital, Nesodden, Norway; ^2^ Department of Rehabilitation Science and Health Technology, Oslo Metropolitan University, Oslo, Norway; ^3^ Department of Child and Adolescent Health Promotion Services, Norwegian Institute of Public Health, Levanger, Norway; ^4^ Department of Psychology, Faculty of Social Sciences, University of Oslo, Oslo, Norway; ^5^ Division of Physiotherapy, Department of Neurobiology, Care Sciences and Society, Karolinska Institutet, Stockholm, Sweden; ^6^ Department of Health and Care Sciences, UiT The Arctic University of Norway, Tromsø, Norway

**Keywords:** rehabilitation, pediatric-onset spinal cord injury, self-management, every day life, coping

## Abstract

**Introduction:**

Pediatric-onset spinal cord injuries (SCIs) significantly impact adolescents' psychosocial and physical developments, posing unique challenges during a critical period of identity formation and progression toward independence. Despite the rarity of pediatric SCIs, the need for understanding how adolescents adapt to their SCIs is crucial. Thus, the aims of this study were to examine and describe the adaption and returning to daily life of adolescents with pediatric-onset SCIs.

**Methods:**

Eight adolescents (4 boys and 4 girls) with SCIs who were aged 11–16 years at the time of the injury were interviewed individually 1–6 years post injury using a semi-structured, strength-based thematic interview guide. The interviews were conducted face-to-face (*n* = 6) or digitally (*n* = 2). A thematic analysis was used to identify key themes in the transcribed data.

**Results:**

Three key themes were identified. Theme 1, “integrating into social life,” highlighted the importance of supportive social networks, with peers and family playing crucial roles. Theme 2, “finding an identity as an adolescent,” underscored the impact of the disability on the adolescent's identity and pursuit of independence. Theme 3, “gaining a sense of control in life,” illustrated strategies for self-care, socializing, and managing physical and psychological challenges.

**Discussion:**

The adolescents in this study who had pediatric-onset SCI demonstrated resilience, adaptability, and agency in navigating social integration, identity formation, and regaining control over their lives. This study emphasizes the importance of social networks and the desire for autonomy in daily life. The participants' experiences suggest an improvement in their involvement in making decisions concerning themselves and a need to inform health-care professionals and improve support for adolescents during and after rehabilitation.

## 1 Introduction

Experiencing a spinal cord injury (SCI) before the age of 18 years (pediatric-onset SCI) is a rare global event, with an incidence rate of 2.9–27 per million/year in Western European countries (1, 2). With the small number of inhabitants in Norway, only 7 cases of pediatric-onset SCI were reported in 2022 and 2023 (3). For those affected, a pediatric-onset SCI is life altering, with lifelong physical and psychological impacts significantly affecting both the affected individuals and their families (4, 5). Moreover, SCIs sustained during childhood and adolescence pose unique and complex challenges, as the injuries occur in a time of mental, physical, and social developmental processes throughout childhood (6).

Depending on its severity and location, the injury leads to paralysis or paresis of either the upper and lower body or only the lower body, with subsequent physical impairments due to loss of sensation, muscle control, and bladder and bowel control. These impairments often require the use of equipment or assistance and may consequently result in dependence on others in daily life activities (7, 8). As such, having a pediatric-onset SCI can be a particularly difficult and challenging situation for adolescents, who are in a phase of transition from childhood to adulthood. Adolescence is a period of life where self-identity and a stable sense of self is developed (9, 10). Adolescents' exposure to other people's opinions about their bodies and appearances may be extra challenging because they are in the process of developing a stable self-identity (8). A previous study that involved 4 school-aged adolescents reported that positive/supportive and negative/rejecting influence from peers impacted their identity and how they perceived themselves (11). In addition, other studies have shown that the experience of shame imposed by how other people perceive adolescents with SCIs may dictate how these adolescents define their body image (12, 13). These studies have shown that adolescents with SCI face unique issues. However, knowledge about adolescents' experiences of engaging and coping in everyday life after SCI is limited. Mulcahey (1992) investigated experiences in managing changes in daily life in the transitional phase after discharge from rehabilitation and returning to school in 4 adolescents with SCIs. Some reported a loss of social contact, whereas others reported gaining new friendships after the injury. The adolescents described returning to school as a difficult transition, and major gaps were identified between the rehabilitation setting and the authentic life at home (11). However, the study included only a small sample and was conducted more than 30 years ago. Although this topic has been explored previously, updated knowledge is needed, given the dynamic nature of societal contexts and the shifts in perceptions of youth and patient-centered rehabilitation. Moreover, enduring an SCI early in life may be accompanied by psychological distress including depression, as highlighted by Klaas (14). They emphasized the need for future research to delve into the resilience of adolescents following pediatric-onset SCIs. Our study aims to contribute to contribute to these knowledge gaps by examining the adaption and returning to daily life of adolescents with pediatric-onset SCIs. Studies have also shown that sleep disturbances, anxiety, pain, and neglect or disregard of the injury and/or health problems are frequent in the adolescent population (12). Betz and colleagues (12) interviewed two adolescents and found the themes “Adjustment to life”, “Hardiness”, “Interdependence” and “Posttraumatic growth”. They found that peers and social networks have a positive impact on the life of adolescents after a pediatric-onset SCI, which advocate for further qualitative research to deepen our understanding of these lived experiences.

In spite of the gross impact of a pediatric-onset SCI, recent studies have indicated that adolescents may have the capacity for positive adjustments to life alterations and SCI-related consequences (15–19). The presence of social support and adaptive coping mechanisms have been identified as important factors for managing SCI-related consequences and adjusting to changes in daily life (20). These have been underscored as factors that facilitate the personal development of adolescents with SCIs, enabling them to maintain quality of life and a sense of purpose that are comparable with those in their preinjury state (21). Resilience is understood as the ability to maintain a stable psychological function and activity in daily life, even after adverse life events (22, 23). It can be used as a theoretical framework to describe and understand the phenomenon of coping and the ability to maintain well-being after a potential traumatic event (24). Bonanno (2021) argued that resilience involves a flexible adaptation to changing circumstances, which allows individuals to bounce back or thrive even after experiencing stress or trauma. Thus, resilience involves both inherent individual resources and abilities, and environmental factors such as support from friends and family (23, 25). Studies have repeatedly shown that resilient trajectories after SCI in adults are the most frequent (26–28). However, research is limited regarding adjustment processes, coping with daily life, and resilience after pediatric-onset SCIs. However, a few studies have discussed that some adolescents with SCIs can exhibit high levels of resilience, good psychological outcomes, and positive coping (29, 30), whereas others have reported the detrimental and life-altering psychological impacts of both minor and severe pediatric-onset SCIs (14). Despite the increasing research on pediatric-onset SCIs, the understanding remains limited regarding how adolescents live with their SCIs and describe their coping with navigating back to their daily lives. Gaining deeper insights into how adolescents manage and adapt to their new realities living with a pediatric-onset SCI can provide valuable information for clinical practice and further research. Thus, the overall aims of the present study were to examine and describe the adaption and return to daily life of adolescents with pediatric-onset SCIs.

## 2 Materials and methods

### 2.1 Study design

The present study is part of a larger project, Phase I of the Sunnaas International Network Pediatric Spinal Cord Injury (SINpedSCI) Project (31, 32). The SINpedSCI project aims to describe the organization of pediatric SCI rehabilitation in 10 rehabilitation units in 7 countries and to examine the psychosocial aspects of living with pediatric-onset SCIs. The present study is based on interview data from the Norwegian part of the SINpedSCI project. An exploratory design was chosen to obtain a detailed description and understanding of adolescents' everyday life experiences with SCI.

### 2.2 Participant recruitment and characteristics

The participants were recruited from a pediatric SCI ward in a hospital for specialized rehabilitation (Sunnaas Rehabilitation Hospital) in Norway. Altogether, 13 adolescents met the inclusion criteria, which were age between 13 and 17 years and having a traumatic or nontraumatic SCI sustained after the age of 7 years, hospitalization at Sunnaas Rehabilitation Hospital during primary rehabilitation, and discharge from rehabilitation at least 6 months before the interview. The exclusion criteria were additional neurological diagnoses that affect cognitive functioning. Most of the adolescents did not respond to written invitations and were thus contacted by phone either by the patient administrator at the hospital or by the first author.

### 2.3 Data collection

**Figure 1:**
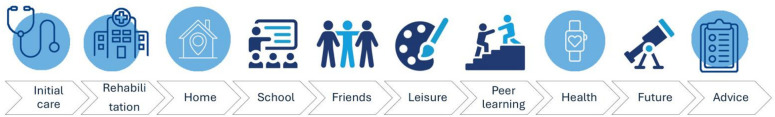
Timeline for the interviews.

Prior to data collection, a thematic semi-structured interview guide with open-ended questions (see Additional File and Figure 1) was developed in cooperation between the researchers and the clinicians with experience in working with children and adolescents. The interview guide was composed of strength-based questions with a salutogenic approach, in addition to established guidelines for psychosocial screening in young persons (6, 32–33). Data were collected from September 2018 to June 2021. The interviews were conducted by 2 female physiotherapists (author W.H., *n* = 3; author V.J., *n* = 5). VJ is a senior physiotherapist and senior researcher who has competencies in pediatric and neurological physiotherapies. W.H. is a senior physiotherapist with competencies in pediatric and neurological physiotherapies. None of the interviewers was responsible for the participants' treatment during their rehabilitation. Each interviewer conducted one pilot interview prior to their first interview.

The interviews were performed either face-to-face (*n* = 6) or on a digital platform (*n* = 2) because of the pandemic. The face-to-face interviews took place either in the participant's home or at the rehabilitation hospital. The digital interviews were conducted on a password-protected online video platform (Norsk Helsenett, The Norwegian Health Web, https://join.nhn.no). The confidentiality of the online interviews was secured by closing the virtual room and providing access only to invited participants. The ethical approval included that adolescents could choose the location for their interview based on what felt most comfortable, they were also asked if they wanted to have a parent present. Some participants opted to conduct their interviews at the rehabilitation hospital during routine check-ups, while others preferred their homes. Additionally, two adolescents chose to have their interviews conducted digitally due to COVID-19, in line with the ethics committee approval. Three adolescents opted to have a parent present during the interview. Injury-related information was self-reported by the participants before the interview started.

A timeline visualizing the time from the accident to the present was used as visual support during the interviews (Figure 1). The timeline covered the main dimensions of adolescent life, including family, school, friends, health, and plans for the future. Within each main area of the interview, key questions were marked to assure that they were asked, and open-ended questions were listed in the interview guide to help the interviewer cover everything. The length of the interviews ranged from 51–121 min, with an average of 72 min. The interviews were recorded with a digital voice recorder and transcribed verbatim. The quotes were translated into English during the writing process, but after we had determined which quotes should be included in the article. The analysis was conducted in Norwegian.

### 2.4 Data analysis

We used a reflexive thematic analysis, which is a flexible method for identifying themes and patterns of meaning in a dataset in relation to the research question (34). In line with Braun and Clarke's (34) method, the analysis involved several phases. First, all the authors read the transcripts to obtain an overall impression. For the coding and analysis of the data in the second phase, we used the digital software program NVivo (35). The first and second authors (W.H. and K.R.) coded the data from each interview. In the third phase, we grouped the codes and created preliminary subthemes and themes related to the aim of the study. All the authors discussed and decided on the code labels and preliminary set of themes. We identified 44 codes altogether. On the basis of the discussions, W.H. and K.R. reviewed and refined the themes before they were validated against the dataset. An analysis was written to capture the story of each theme, describing the adolescents' thoughts and perceptions about their everyday experiences living with a pediatric SCI, as illustrated in Table 1. The themes were then named to provide a sense of what the theme was about (36). The interviews and analysis were conducted in Norwegian.

**Table 1:** Examples of the steps of the thematic analysis, including quotation (sentences and paragraphs), initial codes, and subthemes.

Quotation	Initial code	Subtheme
No, you know, being away from them (friends), it didn't really bother me much; it didn't affect me much because I felt that the closest ones, they were always active on social media. They always made sure I was okay even when I wasn't there. And I appreciate that. Because if they hadn't done that, the situation would have been different. But as I said, when I came back again, it was like I hadn't been away for 2 months, you know.	Being with friends	The importance of others
You have to encourage the person, but don't, you have to do it with sympathy, you have to, you know, you mustn't compare it to anything else. You mustn't think you know how a person feels; you just have to encourage him, with things you know he enjoys. So just say like, “Yeah, let's go out. Yeah, I know you like to be out with friends. Come on, don't sit in there feeling sad. Come out with us. Yeah, okay. So being out with friends helps.”	Don't say you understand my situation, make it better instead	On my own terms
One just has to be good and honest with oneself and make the best out of the situation that has occurred. One just has to make sure that one is as well off as possible in the situation because then there are always people around who support.	Thinking positive	I find something positive with it
Always set a goal you know you won't achieve, set a goal you hope you'll achieve, set a goal you're not sure if you'll achieve or not, and then set a goal within reach. And then you create small goals to reach those ambitions, goals, or dreams you want then.	Set goals for yourself, including ambitious ones	Future perspectives
I would give advice to those young people who have suffered from spinal cord injury. The first thing I would say is “There's always someone who has it worse than you.” And then I would say, “Keep your spirits up. There's always, there's always a way, if your life changes, to find a new way to live life then.” So, it's like getting a spinal cord injury is like changing a morning routine.	Change your thoughts	I handle this.

### 2.5 Trustworthiness

The trustworthiness of the study was maintained by using the Lincoln and Cuba's criteria for credibility, dependability, and transferability, as described by Polit and Beck (37). The credibility was secured by describing the aim and methods of the study and the analysis performed in the study as detailed as possible. Furthermore, we ensured that the informants' voices were preserved by going back and forth throughout the analysis to ensure that the meanings of the codes were always grounded in the original data material. The data were analyzed by 2 authors (W.H. and K.R.) to secure credibility. The analyzed data and extracted codes, subthemes, and themes were discussed with the other authors to ensure the accuracy and interpretation of the findings. The dependability of the study was ensured by transcribing the interviews consecutively, and the interviewers wrote down their impressions of the interview. The codes, subthemes, and themes were cross-checked throughout the analysis. The transferability of the study was secured by providing detailed descriptions of the participants' experiences.

The authors addressed potential threats to trustworthiness, such as the researcher's subjectivity and reflexivity, by discussing their preconceptions before and along with the analysis. The first author is a physiotherapist with experience working with children and adolescents with SCIs. She knew some of the adolescents who participated in the study but were not involved in their therapies. To avoid bias in the analysis of the material, the authors worked systematically to identify biased attitudes and thoughts that represented personal or professional experiences, motivation, and interests (38).

The Consolidated Criteria for Reporting Qualitative Research checklist (39) was used to check and report the quality of this study. We used SIKT KI-chat (https://ki-chat.sikt.no) for proofreading and the translation of the quotes used in this article.

### 2.6 Ethical considerations, data protection, and funding

As part of the SINpedSCI project, the present study was approved by the Regional Committee of Medical and Health Research Ethics of Southeast Norway (2017/1867; 236 December 21, 2017; April 3, 2019, and May 26, 2020). The Norwegian Centre for Research Data (SIKT, formerly NSD) reviewed the study protocol to ensure that it was in accordance with the Personal Data Act and the Personal Health Data Filing System Act (ref. no. 184260). Written informed consent was obtained from the adolescents ≥16 years of age and from the parents of adolescents <16 years of age. In accordance with the Declaration of Helsinki, the information given to the adolescents, both written and verbally, was age appropriate (40). The participants could withdraw at any stage of the study. The data material was stored on a secure server, to which only the project team had access. This study was funded by the South-Eastern Norway Regional Health Authority (project no. 2019124).

## 3 Results

The final sample included eight of 13 eligible adolescents. Four adolescents declined participation due to a reluctance to discuss their injury, and one did not respond to phone calls or text messages. The 8 participants, encompassing 4 boys and 4 girls, were interviewed 1–6 years post injury. Seven participants were paraplegic, and one was tetraplegic. All traumatic injuries were sports related, while the 3 non-traumatic injuries were caused by either a tumor or surgery, shown in Table 2.

**Table 2:** Participants' characteristics.

Participant No.	Age at injury	Age at interview	Etiology
1	13	14	Nontraumatic
2	15	16	Traumatic
3	16	17	Traumatic
4	14	15	Traumatic
5	11	16	Nontraumatic
6	11	14	Nontraumatic
7	14	19	Traumatic
8	15	19	Traumatic

From the analysis, we identified 3 main themes: (1) integrating into social life, (2) finding my identity as an adolescent, and (3) gaining a sense of control in my life. The first theme delves into the adolescents' experiences as they navigated and assimilated into societal interactions and norms during rehabilitation and in the community. The second theme revolves around the adolescents' journeys toward self-discovery and identity formation during this critical phase of their lives. The third theme focuses on adolescents' perspectives on establishing autonomy and exerting control over their own lives.

**Figure 2:**
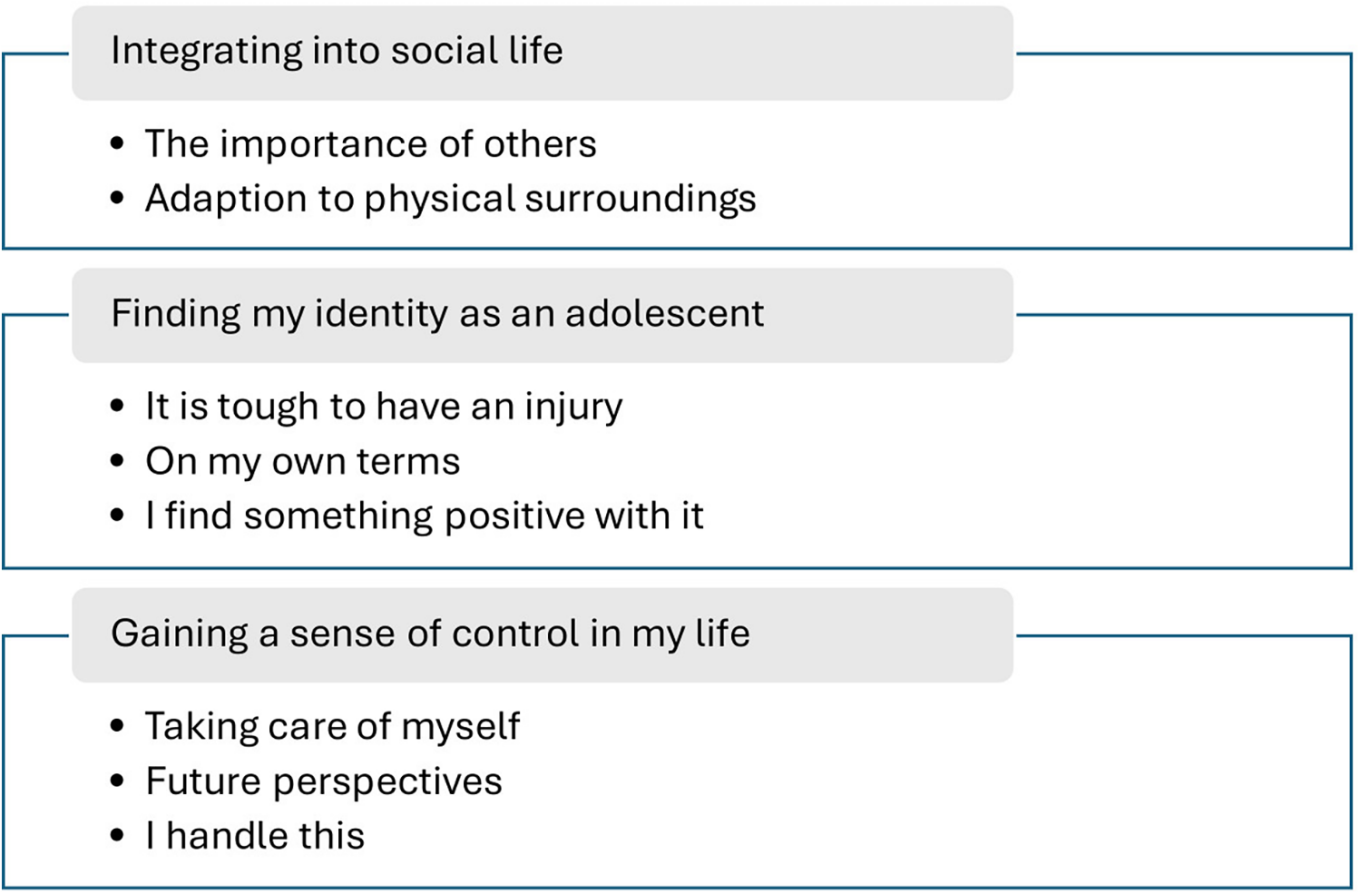
Results of the thematic analysis of the adolescents’ adaption to everyday life with their pediatric SCIs, with the main themes and subthemes.

The themes and subthemes are illustrated in Figure 2. The 3 identified themes encompassed 8 subthemes, which are supplemented with citations from the informants. The quotations are in cursive, and some were edited grammatically to clarify what was expressed. /…/ indicates that some text was left out.

### 3.1 Integration into social life

The theme “integrating into social life” covers the apprehensions and issues faced by the adolescents as they navigated in social interactions within the community, both during and after rehabilitation. The theme delves into the complexities of their social existence and attempts to find a new sense of belonging within their social contexts. Being together with their friends was especially important to all the participating adolescents, both during and after rehabilitation. Having a family that understood their needs and provided practical and emotional support was described by the adolescents as crucial for their growth and ability to succeed in everyday life, as in the following example:

“No, being away from them (friends), it didn't really matter much to me; I didn't care much about it because I felt that the closest ones. They /…/ always made sure I was okay, even though I wasn't there. And I appreciate that. Because if they hadn't done that, then the situation would have been different.” (7)

Despite being away from their friends during rehabilitation, the participants felt reassured knowing that their closest friends ensured their well-being, highlighting the importance of emotional and social support and solidarity within their social circles. This underlines the importance of staying in contact with their closest friends to get back to and reestablish their social lives.

Two participants spoke gratefully about mentors who taught them how to use wheelchairs. One boy's experiences demonstrated the impacts of peer support and mentorship on learning essential skills related to mobility and wheelchair use.

“I talked a lot with X, who is another person with a spinal cord injury. He helped me a lot. He showed me how to do different things and tried to teach me to get in and out of the chair. I failed, but /…/ he and the user consultant have completely taught me wheelchair training and technique. They are the ones who have taught me all the wheelchair techniques.” (4)

Such interactions not only imparted practical knowledge but also fostered a sense of community and understanding among the individuals who were facing similar challenges. However, most adolescents did not express a need for mentors or had any contact with a mentor during rehabilitation.

Several participants highlighted various accommodations made to meet their needs in everyday life. Examples of adjustments were elevators, ramps, wheelchairs, writing assistance, eye-controlled computers, adapted chairs at school, special restrooms, and adaptions in physical education (PE) lessons. The participants considered these adaptions as crucial for enhancing accessibility and facilitating their participation in various aspects of life, such as social activities, school, and sports.

“I got one of those eye-controlled PCs, which kind of became my phone then. /…/ I've used it a lot /…/ in school because I have to write a lot. /…/ It’s very convenient. I do the same on my phone instead of fumbling around; it’s faster if you ask me.” (3)

Moreover, the provision of personalized support and accommodations enabled the adolescents to navigate in social environments with greater ease and confidence. While the adolescents in the sample did not feel excluded by their friends and peers, several experienced being kept out of PE classes at school. Most adolescents did not participate in common PE classes alongside their peers. Instead, they engaged in a specialized program that was individually tailored and supervised by a physiotherapist. One adolescent reported, “*It's okay, I'm excused from PE anyway. So now I don't need to worry about getting a bad grade” (2).* On the other hand, another described that to include her in PE class, her teacher facilitated a set of alternative PE lessons tailored for her: “*If we have parkour, then I have my own setup, for myself, that my gym teacher has arranged. So, he has created a course for me to go through, or I have my own rules. So, if we're playing dodgeball, for example, I won't be tagged on the legs”* (5).

The findings underscore the significance of social adjustments in facilitating the integration of adolescents with pediatric SCI into various aspects of life, showing the adolescents' psychological flexibility and acceptance of important adjustments. Family, friends, school, and peer interactions played pivotal roles in the adolescents' well-being and social integration.

### 3.2 Finding identity as an adolescent along with resilient adaptation processes

The second theme revolves around the adolescents' journeys toward self-discovery and identity formation during this critical phase of their lives. The adolescents reflected on the impacts of their injuries and their self-perception after the abrupt changes in their physical functioning and participation, and how these influenced their personal identities. One adolescent shared that the injury, in spite of the challenges it brought, also led to positive experiences and insights, which contributed to his learning.

“If I had the choice to change it (the injury), I probably would, but at the same time, I think I like this. I've gained a lot of new experiences, met a lot of cool new people, gained some insight here or there.” (2)

This boy acknowledged both challenges and newfound opportunities for personal growth, manifesting his resilience and adaptability in the face of adversity.

The adolescents conveyed a general sense of striving for, or achieving, independence in their everyday lives. They mentioned different strategies for managing their dual roles as persons living with injuries and as adolescents, demonstrating how these experiences shaped their personal identities. One of these strategies was setting goals to push themselves. Their strategies helped them develop both personally, physically, and socially.

“I immediately made up my mind, that I wanted to be able to walk. That was very easy for me to decide, but it depends. If you were very good in sports and wanted to come back to that sport as fast as possible, it is very important that you work hard to reach your goal.” (8)

Being able to manage things on their own was important for all the participants. The adolescents demonstrated a strong desire for independence and self-reliance in their daily lives. From mastering tasks such as getting into a car unassisted to developing strategies for managing challenges associated with their injuries, such as pain or other physical limitations, they exhibited a proactive approach to adapting to their body and navigating their circumstances.

The adolescents also talked about personal development. They described bodily experiences that were influenced by their injuries, such as getting physically exhausted more easily and having more pain. They also experienced mental fatigue when their heads struggled with bearing to engage, especially at school. They needed some time to “come together” and feel like themselves again before they felt they could function at school, as one participant noted: *“No, I… kind of gave up school a bit, in 10th grade… I wasn't available, you know… I would think it had something to do with me getting that injury, and I got a hit, so to speak, that there was something there.” (8)*.

In spite of their complaints, the adolescents asserted their identities as young individuals, not as individuals defined by a disability. They expressed a desire to be seen and treated as adolescents with unique personalities, aspirations, and experiences. The adolescents articulated diverse viewpoints regarding the impact of their injuries on their identities. While unique to everyone, these perspectives collectively highlight the complex interplay between physical health and self-perception and the multifaceted nature of adolescent identity formation in the context of physical injuries.

Another significant concern was the ability to navigate life on their own terms and within their own parameters. The adolescents wanted to be in charge of their lives and to decide how to live and what should be important in their lives. Being autonomous appeared particularly important for their search for self-identity. They expressed a desire for authenticity from peers and adults around them and appreciated it when others demonstrated genuine care by listening to them and making efforts to improve their situation, even if they did not fully understand it. They provided specific examples to illustrate these points, emphasizing the importance of empathy.

“If you're a friend and you're trying to encourage one of your buddies, you shouldn't put yourself in the situation he’s in because you'll never truly know how a person feels when they've broken their back. So, you can't say, ‘I know how it feels, I once broke my finger. I couldn't text on my phone for 2 weeks.’ … You just have to encourage him with things you know he loves.” (2)

While some adolescents expressed frustration or resignation about the challenges they faced, others actively sought out positive aspects and adopted coping mechanisms to navigate through their environment. Whether it was about adopting healthier habits, embracing risk aversion, or finding gratitude in unexpected support and opportunities, they demonstrated resilience and adaptive coping strategies in response to adversity.

“I would think that I can go home. At some point (in the rehabilitation) I can get home. Think about the positive. Try not to think too much about the injury.” (5)

One adolescent described that it is not always easy for health-care professionals to know how to ask adolescents about their thoughts, difficulties, and how they perceive their situation, and he recommended always asking again and not giving up easily when the adolescent's answer is short or evasive. Another adolescent gave advice about how to show care about the adolescent:

“Be there! And don't brag too much of the adolescent. It becomes disgusting and unbelievable at some point. It is enough when you say, ‘Well done’ two or three times.” (4)

The importance of genuine support was emphasized by the adolescents, in addition to the understanding they received from their social circles, including friends, family, and health-care professionals. They valued empathy, active listening, and meaningful engagement from others, particularly in acknowledging the unique challenges they faced and offering support tailored to their needs.

Another topic the adolescents focused on was the positive things in life and the advantages they could draw from their injuries. Two adolescents reflected on how having a SCI influenced them positively, by, for example, eating healthier, working out more and in the right way, and not taking risks, because they had experienced the consequences once. Another participant mentioned that the fact that he was disabled gave him opportunities he would not have had without the disability:

“We've received contributions from the social security system to renovate the bathroom because I am disabled. I would never have done that if I hadn't been injured.” (2)

The adolescents demonstrated responsibility and resilience, and they identified and embraced positive aspects of their injuries and disabilities, even in challenging circumstances*.* In spite of their initial hardships, some adolescents ultimately appeared to find acceptance and even appreciation for the changes brought about by their injuries. They recognized how their experiences shaped them positively, which led to their personal growth, new opportunities, and unexpected blessings. They identified themselves not only as adolescents but also as persons with disabilities. On the contrary, some adolescents wished that the injury had never occurred, describing the negative consequences and challenges associated with the physical pain and bodily changes that they were afraid would intensify as they age.

In summary, the adolescents' reflections on their identity formation in the context of SCI highlighted themes of resilience, adaptation, self-perception, support, and acceptance. In addition to emphasizing the importance of managing the consequences of SCI and reestablishing a sense of normality, their narratives underscore the complexities of navigating adolescence while managing physical challenges and the importance of fostering supportive environments that empower them to embrace their identities and pursue meaningful lives despite their adversities.

### 3.3 Gaining a sense of control in life

The third theme focuses on adolescents' perspectives on exerting control in their own lives and the management of the consequences of SCI. It highlights the adolescents' journeys toward independence and self-determination after experiencing a SCI. The theme encompasses their perceptions of the challenges posed by or resulting from their injuries, physical and mental self-care strategies, and their future outlooks. The adolescents expressed a strong desire to regain control over various aspects of their lives, particularly in managing daily routines and personal care tasks. They experienced a significant loss of control and autonomy after their injuries and during their rehabilitation. Being back home, they highlighted the significance of being able to make decisions independently, including basic daily activities such as determining sleep schedules, hygiene routines, and dietary choices. This emphasis on self-care reflects their efforts to reclaim agency and autonomy in their post-injury lives.

One boy described that life after a SCI was like changing a morning routine: “*Everyone can do it; you just need to do an effort to achieve it”* (8). Many adolescents prioritized physical exercise and healthy lifestyle habits across different environments, including at school and during leisure times, as a means of self-care and empowerment. They viewed self-care not merely as a necessity but as a motivating lifestyle choice that contributes to their overall well-being and preparedness for the future. By taking ownership of their health and fitness, they achieved a sense of control over their bodies. One boy learned a lot about healthy diets and food during rehabilitation, knowledge that he still applied in his life. Taking good care of oneself was described as a lifestyle and as important in both everyday living and preparing for growing older. Most participants discontinued their physiotherapy sessions, opting instead to pursue independent exercise regimens. One boy expressed that he tended to be more cautious than his friends, particularly regarding engaging in risky activities in response to previous experiences.

“I've always been willing to take risks on things, like the reason the injury happened was that I took a chance, you know. /…/ (Sometime after the injury) I was going skiing, and we were with a group of friends, they really wanted to ride the forest trail there, and it’s a bit challenging to do that, so I just said, ‘I'll pass,’ drove around, and met them at the bottom of the hill, because I know that things can happen quickly.” (7)

Despite having various functional limitations, the adolescents described their health as being as good as their friends' health, both physically and mentally. Like most adolescents, the participants in this study wished for a safe and positive future, but they did not want to think too much about what lies ahead; instead, they just enjoyed every day as it came. Several participants adopted a pragmatic approach to managing uncertainty and anxiety. Rather than dwelling on distant or overwhelming possibilities, they emphasized the importance of living in the present and focusing on immediate goals and aspirations.

“Don't think about the future. You don't need to. The future is just awful sometimes. If I think 5 years ahead, then I'll end up in this spiral where I think; oh, now it’s been 5 years of just sitting and not doing anything in a wheelchair. And I just think it’s awful. So, I like to think; tomorrow I'll do this and that. That'll be fun.” (4)

This girl demonstrated how she took control over the negative thoughts that sometimes threatened to occupy her mind, the so-called rumination of negative thoughts. Her strategy was to take one day at a time and think about the nice things she was going to do the next day. By doing so, she gained control of her emotions and kept negative thoughts about the future at a distance.

While most adolescents did not have explicit thoughts about their future, pursuing goals emerged as a powerful strategy for reclaiming control and fostering personal growth and positive hopes for the future. The participants recognized the value of setting both ambitious and attainable goals and breaking them down into manageable steps. They believed that establishing goals was crucial for advancing life and making progress in areas of personal interest. By working toward goals, they put themselves in the front seat, regaining a sense of purpose and direction, which empowered them to navigate challenges and move forward.

“Always set a goal you know you won't achieve, set a goal you hope you'll achieve, set a goal you have no idea if you'll achieve or not, and then set a goal within reach. And then you create small goals to reach those ambitions or goals or dreams you have. /…/ So, then you set small goals, you set a goal you know is within reach, and then you set small goals to get there.” (2)

## 4 Discussion

This study is the first to examine and describe the daily life experiences of Norwegian adolescents with pediatric-onset SCI based on their reflections on their journeys back to everyday life after being discharged from in-patient rehabilitation, with a focus on how they coped with social integration, dealt with identity formation, and regained control in their lives, as well as their adaption and coping strategies. Their transition back to their home environments and adolescent lives is illustrated by 3 themes: (1) “integrating into social life,” (2) “finding an identity as an adolescent,” and (3) “gaining a sense of control in life.” Theme 1 underscores the crucial role of supportive social networks in facilitating community integration. Theme 2 addresses concerns regarding the impact of disability on adolescents' identity and the importance of maintaining independence in daily activities. Theme 3 outlines the adolescents' strategies for physical and psychological self-care, highlighting the significance of social interactions, coping mechanisms, exercise, diet, and risk assessment.

### 4.1 Resilience and coping

Resilience is an asset and a crucial factor for adolescents with SCI in managing life after rehabilitation. It is described as a pattern of functioning that promotes effective coping with adverse life events (41). Some of the adolescents interviewed in the present study maintained the ability to focus on the positive aspects of their lives, in spite of having negative thoughts and feelings such as anger and sadness about and the consequences of their injuries. Most adolescents showed a proactive mindset and adaptive, effective coping strategies such as seeking emotional and instrumental support and exhibiting a fighting spirit (i.e., efforts to act independently). Emotional support was primarily provided by family, friends, health-care staff, and teachers. The adolescents described various ways of positive coping and the ability to adjust to living with SCI-related consequences. This finding adheres to studies that showed that resilient trajectories after SCI are the most common trajectories in the adult population (22, 27, 41). At the same time, the adolescents reflected on their injuries as both a blessing and a burden but one that they could most likely handle. This could mean that they have individual resilience in dealing with SCI consequences and finding adjusted ways of engaging in daily living and social life. This also reflects an openness to accept change and derive meaning and value in adversity, which ultimately enhance resilience and well-being. This coheres with acceptance, a coping strategy identified by Anderson et al. (42) and that has been found to be particularly evident in adult individuals with SCI and those who had sustained injuries for a long time (42). In this context, acceptance means accepting the fact that an injury occurred and learning to live with it.

Several researchers (20, 43, 44) have investigated resilience in adolescents with SCIs, revealing various coping strategies and efforts, showing that many adolescents employ their abilities to bounce back and maintain psychosocial functioning (43). This includes seeking social support, reframing perspectives, setting goals, and engaging in meaningful activities (43). The findings coincide with those of the present study. The participants described how they managed everyday life and shared some of their methods going forward, such as setting goals, both small and reachable and more demanding ones, and looking for joy every day.

Thus, our findings correspond with those of other studies that identified important factors that contribute to resilience in adolescents with SCI, such as social support, positive coping strategies, self-efficacy, psychological well-being, and environmental factors (45–47). The environmental factors underlined in the present study include support from peers, family, and health-care professionals; technical assistive devices; and involvement in activities.

### 4.2 Social support and participation

Our findings highlight the significance of supportive environments and personalized accommodations to facilitate adolescents' participation and well-being. Mohan and Deb (2024) found similar results in adults with SCI in India. Our interviews showed that after the injury, the adolescents made social adjustments that significantly impacted various aspects of their lives. Some adolescents' comments indicated that they felt included in some contexts, such as on digital platforms, as a part of the social society with their peers. However, they also noted feeling more exposed in social settings owing to their injuries, which sometimes led to their being treated differently. Previous studies have also identified social support as an important factor for adolescents with SCI to be able to adapt positively after their injury (20, 48). As demonstrated in previous studies (12, 49), peer support can have a positive impact on both the physical and mental health of individuals with SCI. Building on these findings, the present study emphasizes the need for empathy and understanding from peers, family, and health-care professionals in supporting adolescents on their journey toward independence and self-actualization. Health-care professionals also play an important role in adolescents' adaption and self-acceptance (50). Early in the rehabilitation process, they should include adolescents in making decisions that affect them.

Most of the adolescents in our study did not participate in PE classes at school but either received tailored sessions with a physiotherapist (individually) or were exempted from class. Our clinical experiences have shown that young persons with disabilities are frequently excused from PE classes and field trips instead of receiving sufficient environmental accommodations and activity modifications. Hence, the level of participation is influenced by the environment and the adaptability in the environment, both in school and society (51). This highlights the importance of accommodating diverse physical abilities within educational settings and raises concerns about the potential social exclusion or stigma associated with deviating from mainstream activities. Physical activities at school have been emphasized as crucial for social, psychological, and physical health and well-being (52). By acknowledging and addressing the diverse needs of these adolescents, inclusive environments that enhance their overall well-being and participation in society should be endeavored.

### 4.3 Identity formation

During adolescence, a critical aspect of psychosocial development is the formation and consolidation of personal identity (53). Major challenging life events in adolescence, such as incurring a SCI, may influence the development of identity and cause changes in psychosocial development (53). Our study highlights the importance of adolescents' sense of agency and autonomy in shaping their own positive identities and narratives. The adolescents in the present study conveyed that it was important for them to live and to be perceived and treated as “normal” adolescents like their friends and peers. On the other hand, some adolescents appreciated the benefits they received from being a person with a disability, such as receiving financial for the construction of a new bathroom in their house. They expressed appreciation for the opportunities they received because of their disabilities. This demonstrates that adolescents with SCIs may identify with multiple roles and identities, that is, being a regular adolescent with ideas and values similar to those of able-bodied peers and identifying oneself as a young person living with a disability. This is in line with a recent study by Forber-Pratt et al. (54) that showed that adolescents with various disabilities expressed heterogeneous perspectives about disability identity. Their perception of disability identity depended on their social environments and general life experiences, both before and after the injury (54). Bicultural identities, or multiple identities, have been introduced in the literature (55, 56) to describe how identity can be both personal and social (55, 56). The adolescents in this study described bicultural identity as both a “normal adolescent” and an “adolescent with a disability,” depending on the social setting. Maintaining one's identity requires having people around who care and confirm that one is a social being (55). The adolescents emphasized the importance of fostering supportive environments that validate their experiences and empower them to thrive in their daily lives. Dunn and Burcaw (57) indicated that disability identity includes a desire to be recognized and treated like everyone else in the group or society. Continuing normal activities with peers, such as work, sports, and social activities, has been highlighted as crucial for fostering a sense of positive self-worth (57).

### 4.4 Regaining control in their lives

The importance of taking care of themselves, being autonomous, and being in control of their actions and lives was highlighted by some adolescents in the present study. Adolescence is a complex developmental phase in which behavior patterns are established, such as emancipation from parents, learning about one's own body, developing sexuality and function, and interacting socially (58). In the present study, the adolescents demonstrated a nuanced approach to risk management and decision-making, informed by their past experiences and the consequences of taking risks. For example, one adolescent who was injured after a skiing accident described taking increased caution compared with his peers when engaging in risky activities, which in his case was off-piste skiing. This proactive mindset allows adolescents to maintain control over their lives and emotional well-being. Similarly, Betz et al. (12) found that adolescents with SCIs took responsibility for their choices to find effective solutions in challenging situations.

Another aspect that the adolescents discussed was the importance of setting goals to make progress in life personally, academically, and professionally. Maribo et al. (59) concluded that individuals with SCIs often set goals for everyday life during rehabilitation. The need to be involved in the goal-setting process to pursue their goals was emphasized by adult rehabilitation participants (60). Although goal setting is a crucial component in rehabilitation and is often primarily initiated by health professionals (61), the adolescents in the present study appeared to apply knowledge in goal setting to their everyday lives. Through their reflections on goal setting, risk management, and lifestyle choices, the adolescents demonstrated adaptive strategies for coping with the uncertainties and challenges posed by their injuries. These skills align with the principles of self-management, crucial for adolescents with pediatric-onset SCIs, as they navigate both life and the healthcare system (62).

By focusing on what they can control and actively shaping their futures, the adolescents appeared to regain control over their lives and cultivate resilience and agency in the face of adversity.

The findings from this study have the potential to impact the clinical practice of healthcare professionals improving rehabilitation for children and adolescents with pediatric-onset SCIs. A key consideration is to include the adolescents actively in all decision-making processes concerning their rehabilitation, education, and life after rehabilitation. In this way, they are given responsibility and autonomy in their lives. Additionally, maintaining social connections and minimizing hospital stays are critical in preventing social and academic isolation (63).

This study specifically examines adolescents with pediatric-onset SCIs, a group facing unique challenges due to the early onset of their injury. Unlike those who acquire SCIs later as young adults, these individuals must navigate key developmental stages while already living with their injury. These challenging adaptation process influence identity development, independence, and social participation. While some findings may apply broadly to adolescents with SCIs, this study underscores the distinct long-term adaptation processes of those with pediatric-onset SCIs, contributing to a deeper understanding of their experiences.

This qualitative study primarily highlights the challenges and barriers faced during adolescence; these challenges probably started already at the onset of their injury. Adaptations made during adolescence build on an ongoing process that starts much earlier. Future research should examine these earlier stages to provide a more comprehensive understanding of the full adaptation process.

### 4.5 Strengths and limitations of this study

The equal number of male and female adolescents included in this study is a strength. The longest time since injury was 5 years, which might have led to diminished detailed memories from the time immediately after the injury and of how they thought and reacted. However, the strength of this study is the variability in the answers and perspectives of those with older and those with recent injuries at the time of the interview. The transferability of the findings may be limited, as they represent the views of a small sample of 8 Norwegian adolescents living at home after rehabilitation. Nevertheless, it can be argued that information power was achieved because the cohort provided substantial insights relevant to the study (64). For qualitative research, it is argued that participants should reflect the diversity of the culture and conditions, including race. Regarding the present study, all the participants were ethnic Norwegian, making this a potential limitation for the transferability of the study to an international context and different ethnic backgrounds. However, we gained much learning from these adolescents that can be applied to international rehabilitation efforts, given their universal demands and needs as adolescents. The adolescents in this study appeared quite empowered and well-functioning.

Children and adolescents have a legal right to have a parent or guardian present during interviews (63). The interviewers acknowledged the potential bias and addressed this by using interview techniques and their profound knowledge about pediatric-onset SCIs. Though, the presence of a parent may have influenced the conversation and the information the adolescents choose to share with the interviewer.

A limitation of this study is the low representation of participants with a tetraplegic injury, which may have constrained the variation in experiences captured in this article. Given that individuals with different levels of SCI often face distinct challenges, including additional participants with tetraplegic injuries of different genders could have provided greater diversity in perspectives. The results shown are mostly regarding the psychological and mental state of the adolescents, and they do not focus that much on the physical affection of the injury. However, the main themes identified in the study were shared across participants with varying injury levels, suggesting that the overall findings remain relevant. This limitation highlights the need for further research with a larger and more diverse samples, considering different injury levels and genders.

## 5 Conclusion

The aim of this study was to examine and describe the adaption and reintegration into the daily lives of adolescents with SCI after in-patient rehabilitation. The findings underscore the resilience and significance of both internal and external factors in fostering resilience, adaptability, and agency that these adolescents demonstrate as they navigate the complexities of social integration, identity formation, and regaining control over their lives. This study emphasizes the importance of supportive social networks in adaptive post-injury integration. In addition, it concludes that health-care professionals, parents, teachers, and others must involve adolescents in making decisions concerning themselves from the very first day of the onset of the SCI.

This is the first study to examine the post-rehabilitation daily lives of Norwegian adolescents with pediatric-onset SCI, with a focus on their adaption and coping strategies. By understanding and addressing their diverse needs, we can create more inclusive environments that support their overall development and integration into society. It is important for future studies and interventions to aim at enhancing the qualities of life of adolescents with SCIs and understanding adolescents' needs and desires. Further qualitative research is needed for a more in-depth understanding of the psychosocial factors that affect young people with SCI and the complexity of coping. The findings of our study provide valuable insights into the multifaceted experiences of adolescents with SCI as they navigate social interactions, peer support, accessibility, and educational accommodations.

## Data Availability

The raw data supporting the conclusions of this article will be made available by the authors, without undue reservation.
